# Post retraction citations among manuscripts reporting a radiology-imaging diagnostic method

**DOI:** 10.1371/journal.pone.0217918

**Published:** 2019-06-13

**Authors:** Sorana D. Bolboacă, Diana-Victoria Buhai, Maria Aluaș, Adriana E. Bulboacă

**Affiliations:** 1 Department of Medical Informatics and Biostatistics, Iuliu Hațieganu University of Medicine and Pharmacy, Cluj-Napoca, Romania; 2 Faculty of Medicine, Iuliu Hațieganu University of Medicine and Pharmacy, Cluj-Napoca, Romania; 3 Department of Abilities and Human Sciences, Iuliu Hațieganu University of Medicine and Pharmacy, Cluj-Napoca, Romania; 4 Department of Pathophysiology, Iuliu Haţieganu University of Medicine and Pharmacy, Cluj-Napoca, Romania; KU Leuven, BELGIUM

## Abstract

Our study aimed to evaluate the trends of post retraction citations of articles reporting a radiology-imaging diagnostic method and to find if a different pattern exists between manuscripts reporting an ultrasound method and those reporting other radiology diagnostic methods. This study reviewed retractions stored in PubMed on the subject of radiology-imaging diagnosis to identify the motivation, time from publication to retraction, and citations before and after retraction. The PubMed database was searched on June 2017 to retrieve the retracted articles, and the Scopus database was screened to identify the post-retraction citations. The full text was screened to see the type of post-retraction citation (positive/negative) and whether the cited article appears or not as retracted. One hundred and two retractions were identified, representing 3.5% of the retracted articles indexed by PubMed, out of which 54 were included in the analysis. Half of the articles were retracted in the first 24 months after publication, and the number of post retraction citations was higher than the number of citations before retraction in 30 out of 54 cases (US methods: 9/20, other diagnostic methods 21/34, P-value = 0.2312). The plagiarism was the most common reason for retraction (31%), followed by repetitive publication (26%), and errors in data/manuscript (24%). In less than 2% of cases, the retracted articles appear as retracted in the text or reference list, while the negative citation is observed in 4.84% among manuscripts reporting an US diagnostic method and 0.32% among manuscripts reporting a diagnostic method other than US (P-value = 0.0004). No significant differences were observed when post retraction weighted citation index (WCI, no. of citations weighted by citation window) was compared to WCI prior retraction (P-value = 0.5972). In light of the reported results, we enumerated some recommendations that could potentially minimize the referral to retracted studies as valid.

## Introduction

Research is the primary source of knowledge in medicine and publication is the principal instrument for dissemination of research results. Furthermore, scientific productivity in academia is traditionally assessed based on the number of publications. Consequently, new scientific journals appear even if some of them are classified as ‘predatory’ (lacking integrity in the publishing process usually without an accurate peer review process, fake impact factor or editorial team, issues that could be difficult to detect) and the number of published scientific articles increases annually [1–3]. The retraction of an article is used to alert scientists to serious problems identified with a published article and follows the Committee on Publication Ethics retraction guideline [4]. According to this guideline [4], a retraction should be considered if clear evidence exists that ① the findings are unreliable because of misconduct (such as data fabrication, data falsification, etc.) or ② honest errors (such as experimental error, or miscalculations), ③ the findings have previously been published (redundant publication), ④ the text constitutes plagiarism or ⑤ the article reports unethical research. The retraction notice should be linked to the retracted article and clearly labeled by including the title and the authors in the retraction heading, designating the retraction, and specifying a clear reason for retraction [4,5].

Honest error or mistakes, non-replicable findings, research misconduct, and redundant or duplicate publication are the main reasons for retraction [6–8]. The term “honest error/mistake” is frequently used, but it is not clear how journals or publishers establish if an error was honest or purposeful [9]. The reported reasons for retraction are slightly different among medical specialties but misconduct, plagiarism, duplicate publication, or authorship issues are most frequently reported (orthopedics [10], dentistry [11,12], neurosurgery [13], cancer research [14], emergency medicine [15], radiology [16], nursing and midwifery research [17]). Systematic manipulation of the peer review processes by ensuring that a specific article passes the peer review in return for a fee paid by the authors, or for boosting their own publications, and inappropriate authorship, including offering authorship of the article in return for a fee are lately reported [18–20].

The term “retraction” is redefined and new words are introduced to reflect the reason and/or the person who retracted the paper(s) [21]. *Retraction* is used for misconduct, *withdrawn* for paper(s) retracted by author(s) due to new evidence, data, methodologies, results, etc. that abolish the stated claims, *retired* to identify outdated guidelines, *canceled* for retraction due to an editorial, production or publishing mistake, *self-retraction* for retractions signed by all co-authors, and *removal* to identify the papers with content presenting a risk to society, individuals, or to the environment.

Rosenkrantz conducted an analysis of the retracted articles published in radiology journals [16] and identified the retraction as uncommon (1.1% of all PubMed "retracted publication"). The retraction notification was available in most of the cases (39/48) and the retracted articles received post-retraction citation (mean±standard deviation = 5.2±12.0, median = 3, range from 0 to 70) [16]. Hong et al. reported an overall rate of duplicated publication in radiology journals of 1.92/1,000 citations (ranging from 0 to 10/1000) and a sensitivity of iThenticate equal with 61.9% for “possible duplicates” (defined as a matching of more than 30%) [22]. Plagiarism in manuscripts sent for publication to the American Journal of Roentgenology was reported in 12/110 articles scanned, most frequently identified in the Abstract, Materials and Methods, and Discussion sections [23]. Honorary authorship in radiologic research articles (overall, 26.0%) is reported by low academic rank researchers (OR = 2.89, 95%CI [1.66 to 5.06]), working in settings where the head of the department must be listed as an author (OR = 3.80 [2.13 to 6.79] [24]. The suggestion of adding an author / co-authors who performed non-authorship tasks determined the honorary authorship in most cases [25]. The honorary authorship is more frequently seen in Asia and Europe as compared to North America and in research environments where the head of the department is automatically listed as co-author [26].

Previous research was conducted on radiology journals, but articles reporting a radiology-imaging diagnostics method are published in any medical journals. Retracted articles are not expected to be cited, or the citation must include both the original manuscript and the retraction notice, with clear notice of retraction at least in the reference list. To the best of our knowledge, the trend of citation after retraction among articles reporting on radiology diagnostic methods has not been systematically evaluated. For that reason, we aimed to identify and quantify the citation before and after retraction of articles reporting a radiological diagnostic.

## Materials and methods

PubMed database, involving PubMed Clinical Queries was searched to identify retracted articles using the following keywords: (Diagnosis/Broad[filter]) AND ((diagnostic or diagnosis) and (imaging or radiology)) AND ((Retracted Publication[sb] OR Retraction of Publication[sb]) AND Humans[Mesh] AND English[lang]))). No limit of years was imposed, and we only looked after publications in English that reported research on humans. The PubMed Clinical Queries was searched on June 15, 2017. The retrieved documents were first screened by reading the title and the abstract. The abstract was read from PubMed and, whenever available, the journal web page was screened to retrieve the abstract. Articles presenting a diagnostic-related technique such as radiography, radioscopy, ultrasonography (US, all types), computer tomography (CT), magnetic resonance imaging (MRI), positron emission tomography (PET), single photon emission computed tomography (SPECT), or scintigraphy were included in the analysis. The following data were collected for each publication included in the analysis: year and month of publication, the reported diagnostic imaging method, the journal, no. of authors, the authors’ affiliation country, month and year of retraction, the reason for retraction, number of citations until and after retraction. The Journal Citation Reports was used to retrieve the domain and the rank of the journal whenever applicable. The time expressed in months from publication to retraction and from investigation to retraction was calculated based on the collected data. The citation pattern is influenced by many factors, the age of the paper and the citation time windows being the deciding factors [27–29]. The weighted citation index (WCI) was defined as the ratio between the number of citations and the citation time window (no. of months/12). The WCI was calculated both prior to (WCI_prior_) and post (WCI_post_) retraction to analyze the dynamic of citations.

The title of each retracted manuscript was searched in the list of references using the Scopus database to identify the citations. The search was conducted in September 8^th^, 2017 and the number of citations prior to and post retraction (according to month and year) was recorded. The link to the paper citing a retracted manuscript was also stored for further processing, gathering all post retraction citations on articles indexed by Scopus until September 8^th^, 2017. To analyze the type of post retraction citation (positive/negative) and to identify whether an explicit notice concerning the retraction status of the cited article (yes/no) exists in the body of the article or in the reference section, the full text of the papers citing a retracted manuscript were screened. The full-text screening of papers citing a retracted manuscript was done from 9^th^ September to 22^nd^ December 2017, on all titles identified on September 8^th^, 2017.

The collected data were used to characterize the retracted publication (such as type of the manuscript, journal rank from the Journal Citation Reports (JCR) for Web of Science (WoS) indexed journals, number of authors, time between publication and retraction, the reason for retraction, citation prior to and post retraction, etc.), summarizing the characteristics for the whole sample or for those articles that reported an ultrasound related diagnostic (US). Frequencies as absolute (number) and relative (percentage) with an associated exact 95% confidence intervals [30] were used to summarize the data. Median and interquartile range were used to report the number of authors (of the retracted article itself and of the article that cited a retracted manuscript), number of months between publication and retraction, citations (prior to and post retraction), and WCIs (since data proved to be significantly different by the theoretical normal distribution: Kolmogorov-Smirnov test, p<0.05). Graphical representations were drawn with Microsoft Excel. Mann Whitney test was used to compare the time from publication to retraction between articles that reported ultrasound diagnostic techniques compared to those reporting other radiology diagnostic methods. The difference between WCI prior to and post retraction were tested using the Wilcoxon Pairs Test. The association between the type of citation (positive/negative), higher WCI_post_ as compared to WCI_prior_ and reported diagnostic technique (a US method/other than US method, where US = ultrasound) was tested with Chi-square test. The significance level was set at 0.05, thus all P-values less than 0.05 were considered statistically significant.

## Results

**Fig 1:**
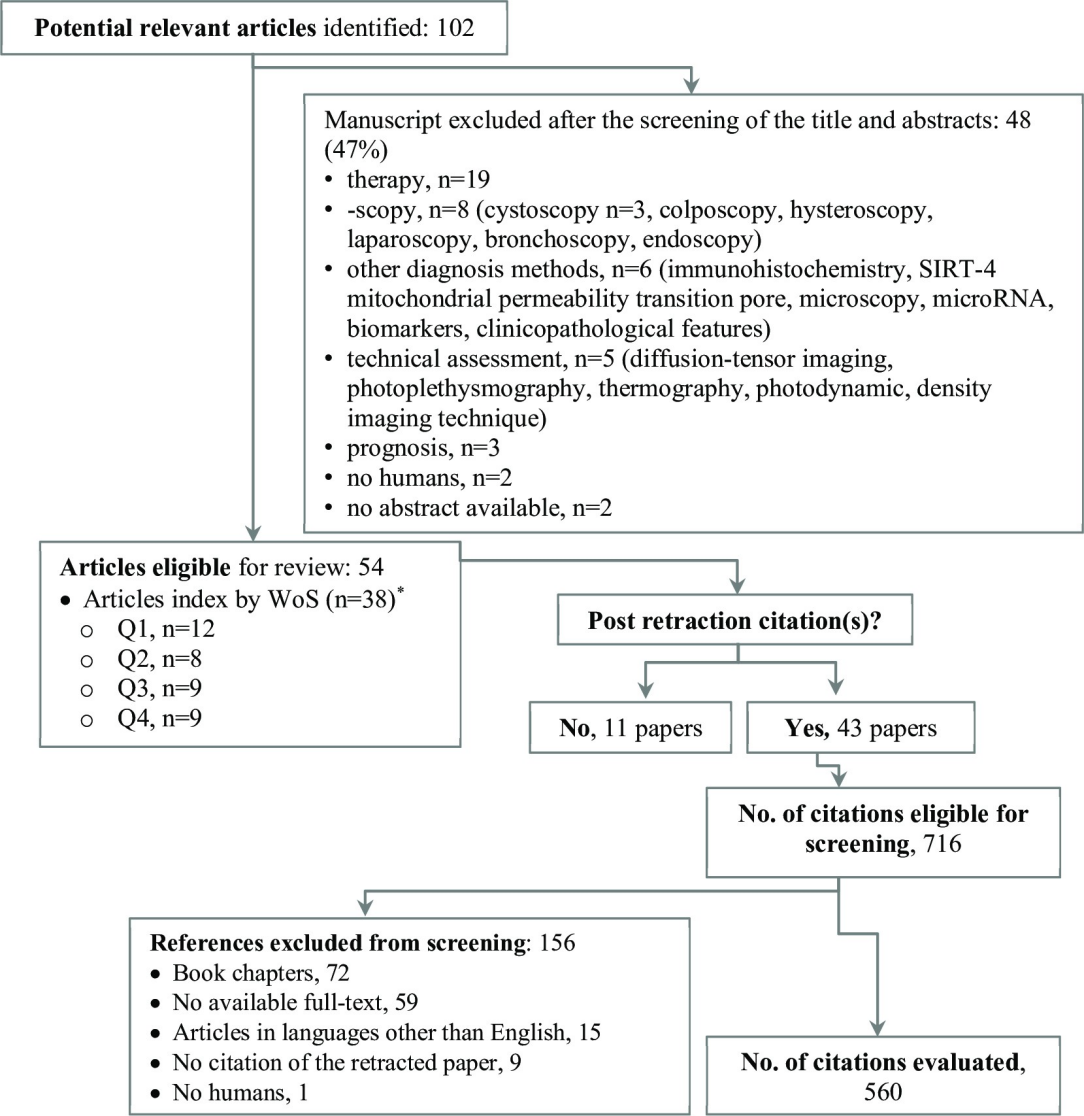
Flowchart of screened manuscripts and reason for exclusion. The search included only studies with an available abstract on PubMed or on the web page of the journal. *The retracted articles indexed by WoS were published in journals classified into the following domains: *Radiology*, *Nuclear Medicine & Medical Imaging* (11 articles), *Peripheral Vascular Disease*, *Clinical Neurology*, *Cardiac & Cardivascular Systems* (each with 4 articles), *Medicine*, *General & Internal* (3 articles), and *Urology & Nephrology* (2 articles). All other WoS domains had one retraction (*Anesthesiology*, *Dentistry*, *Oral Surgery & Medicine*, *Emergency Medicine*, *Endocrinology & Metabolism*, *Mathematical & Computational Biology*, *Ophthalmology*, *Orthopedics*, *Pathology*, *Pediatrics*, *Surgery*).

A total of 2,897 articles indexed as a publication type of “retracted of publication” or “retraction of publication” published until June 15, 2017, on human species and in the English language were identified under the Diagnosis Clinical Study Categories, PubMed Clinical Queries. One hundred and two articles (3.5%) were retrieved for the searched keywords, and 54 were finally included in the analysis. Forty-eight articles were excluded and the reasons for elimination are given in Fig 1.

**Fig 2:**
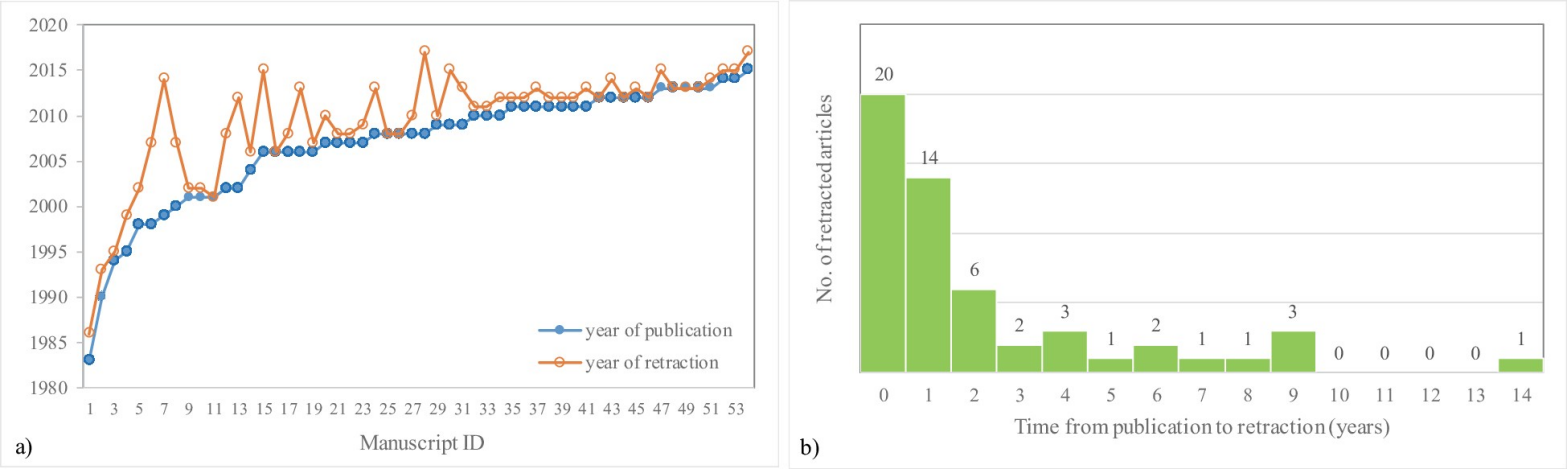
Retractions trends. a) Year of publication and year of retraction; b) The number of years from publication to retraction. Zero in graph b) means that the article was retracted in the first 12 months after publication.

The earliest retracted manuscript was published in 1983 (retracted in 1986) and the latest in 2015 (retracted in 2017) (Fig 2A, S1 File). The highest number of retractions was observed in 2012 for the whole sample (10/54, 19%), as well as for those that reported US techniques (4/20, 20%). In most cases, the retraction took place in the first 12 months after publication (Fig 2B). The time (in months) between publication and retraction is significantly higher for articles reporting an US diagnostic method (Mann-Whitney test: statistic = -2.95, p = 0.0031). The characteristics of the retracted manuscripts are presented in Table 1.

**Table 1:** Characteristics of the retracted manuscripts.

	Whole sample (n = 54)	Ultrasound manuscripts (n = 20)
Type-of-manuscript, no (%)		
case report	10 (19)	3 (15)
case series	6 (11)	2 (10)
research article	34 (63)	15 (75)
review	4 (7)	0 (0)
JCR journal rank, no (%)		
Q1	12 (22)	4 (20)
Q2	8 (15)	4 (20)
Q3	9 (17)	2 (10)
Q4	9 (17)	3 (15)
Non indexed in WoS	16 (30)	7 (35)
No. of authors, median (Q1 to Q3)	6 (3 to 8)	6 (4 to 9)
Months from publication to retraction		
Median (Q1 to Q3)	16 (9 to 37)	32 (15 to 82)
Reason for retraction, no (%)		
duplicate/repetitive publication	14 (26)	5 (25)
errors in data/manuscript	13 (24)	3 (15)
plagiarism	17 (31)	7 (35)
publisher error	4 (7)	1 (5)
inappropriate peer-review	1 (2)	1 (5)
no consent of the co-authors	2 (4)	0 (0)
no motivation	3 (6)	3 (15)

Numbers are absolute frequency with column percentages in parentheses.

JCR = Journal Citation Reports–Clarivate; WOS = Web of Science

The number of authors varied from 1 to 12, without significant differences when US manuscripts are compared to the manuscripts reporting other radiological diagnostic methods (Mann-Whitney Z statistic = 0.60, p = 0.5485). All retracted manuscripts had authors from one country. The highest number of authors have the affiliation in Germany (12 authors), followed by USA and Romania (each with 11 authors), Italy, France, and Netherlands (each with 10 authors). Most of the retracted articles had authors from the USA (7/54, 13%), followed by Japan and India (6/54, 11%), and China (4/54, 9%).

Considering the manuscripts reporting an US diagnostic method, the highest number of retracted articles are from Italy (3/20, 15%), and respectively India, Germany, and Croatia (each with 2 retracted articles, 10%).

*Acta Radiologica* and *Croatian Medical Journal* were the journals with two retracted manuscripts. The journal with the smallest frequency of retraction was *American Journal of Roentgenology* (0.0038%, WoS domain as *Radiology*, *Nuclear Medicine & Medical Imaging*). In contrast, the journal with the highest frequency of retraction was *Diagnostic Pathology* (4%, WoS domain as *Pathology*).

**Fig 3:**
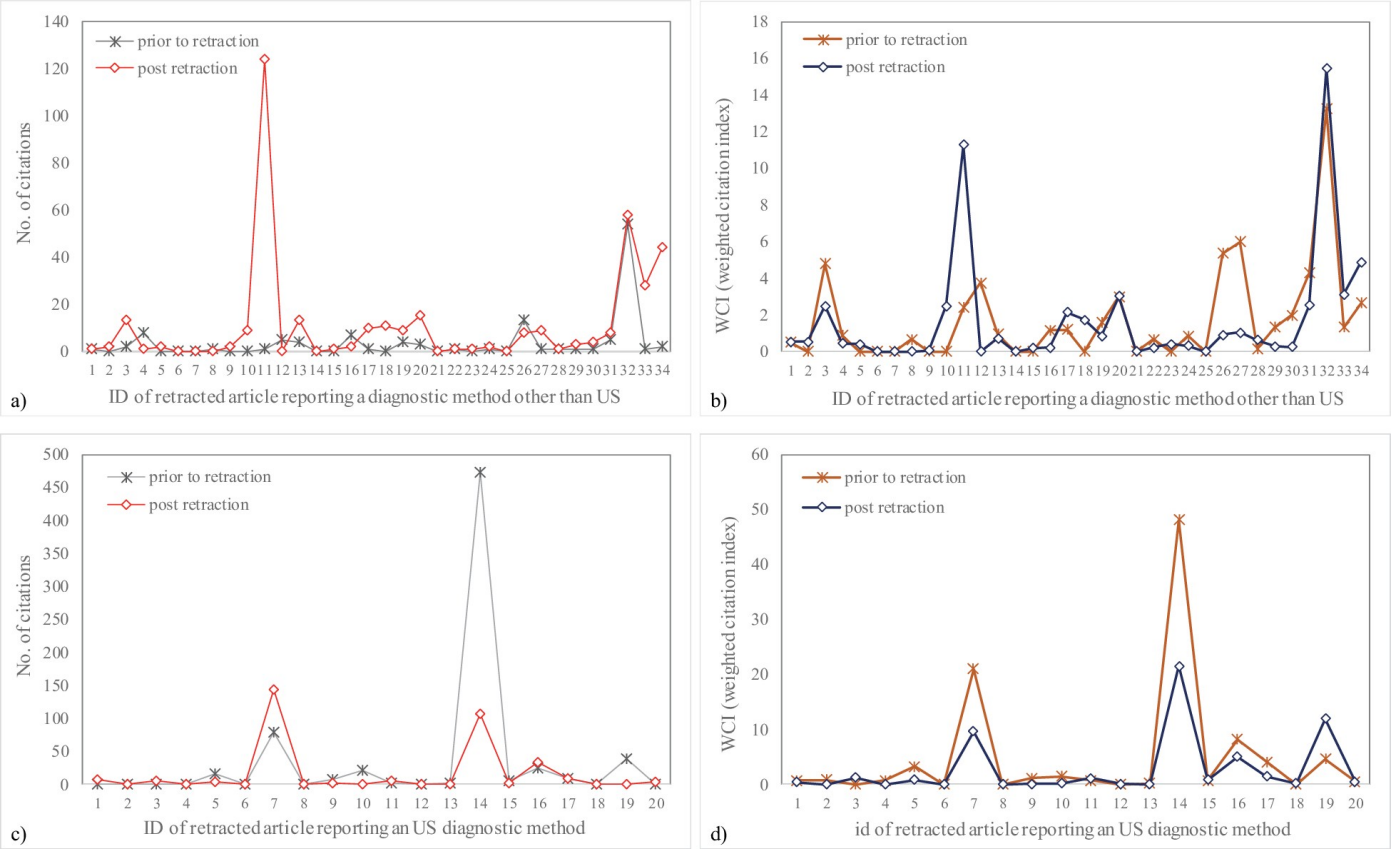
Retracted articles that reported a diagnostic method. Other diagnostic methods than US: a) Absolute number of citations, b) Number of citations weighted by citation window. US diagnostic method: c) Absolute number of citations, d) Number of citations weighted by citation window.

Thirty-seven out of fifty-four investigated papers had at least one citation prior to retraction and the highest number of citations (473) is of a duplicate publication issued in another journal in the same year, but retracted almost 10 years after publication (Fig 3). The number of citations post retraction varied from none to 144 (the highest number was registered for a paper published in 1998 and retracted in 2002 due to the unmistakable similarity of one figure). The number of citations post retraction was higher compared to the number of citations prior to retraction in 30/54 cases (US methods: 9/20, other diagnostic methods 21/34) but with no significant association with the reported diagnostic radiology method (Chi-square test: 1.43, P-value = 0.2312).

The WCI_prior_ index varied from zero to 48.10 (a duplicate paper with 473 citations prior to retraction in 118 months) with no significant differences when the papers reporting US diagnostic methods were compared to those that reported other radiology diagnostic methods (US: 0.81 (0.16 to 3.52), other methods than US: 0.87 (0.00 to 2.30), Mann-Whitney Test: P-value = 0.5972). The same pattern is observed on the WCI_post_ without significant differences between papers reporting US diagnostic methods (0.53 (0.10 to 1.35)) as compared with the papers covering other diagnostic methods (0.50 (0.21 to 2.05); Mann-Whitney Test: P-value = 0.9857). No significant differences were observed when WCI_prior_ and WCI_post_ were compared, neither for papers dealing with an US diagnostic method (Wilcoxon Pairs Test: P-value = 0.0778), nor for papers reporting on other diagnostic methods (Wilcoxon Pairs Test: P-value = 0.7375).

The main characteristics of the papers citing a retracted article are shown in Table 2 (raw data available in S1 File).

**Table 2:** Post retraction citations: Characteristics.

	Whole sample (n = 559)	Manuscripts reporting an ultrasound diagnostic method (n = 248)
Affiliation in the articles that cited a retracted paper		
One country no. (%)	502 (90)	221 (89)
More than one country no. (%)	57 (10)	27 (11)
No. of authors, median (Q1 to Q3)	5 (3 to 8)	5 (3 to 8)
Labelled as retracted manuscript (text/references), no. (%)	6 (1.07)	3 (1.21)
Negative citation, no. (%)	13 (2.33)	12 (4.84)
WCI_post_	0.51 (0.14 to 1.70)	0.53 (0.10 to 1.35)

The WCIpost (weighted citation index post retraction) and No. of authors are expressed as median and (Q1 to Q3), where Q1 is the first quartile and Q3 is the third quartile; All other variables are expressed as absolute frequency with column percentages in parentheses.

Most frequently, the WCI_post_ is higher than the WCI_prior_ with regard to retracted papers that reported other diagnostic methods than US (44% vs. 35%, P-value = 0.5184).

**Fig 4:**
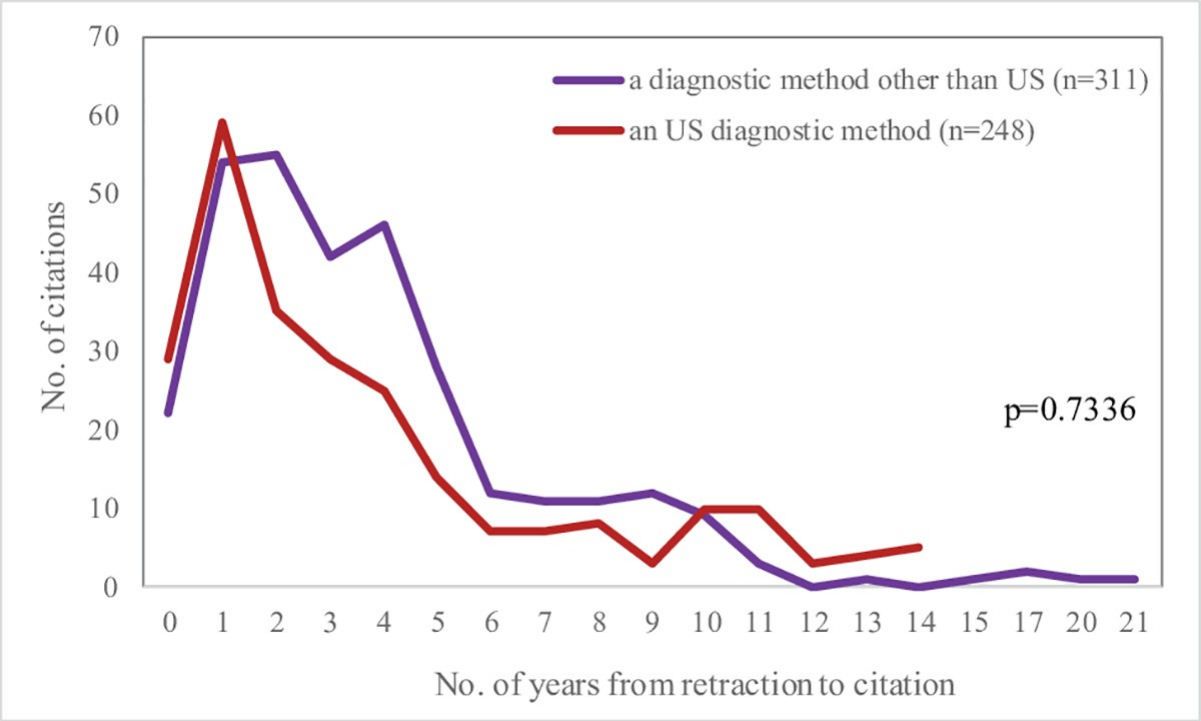
Flow of post retraction citations according to the number of years of the retracted paper. Zero corresponds to the number of citations in the same year when the paper was retracted.

A retracted article is cited up to 21 years, with 58% of citations post retraction in the first four years after retraction, with similar pattern among papers that reported an US diagnostic method as compared to those that reported other radiology diagnostic methods (Fig 4).

The retracted papers reporting an US diagnostic method received a significantly higher number of citations (35%) as compared to the retracted papers reporting diagnostic methods other than US methods (24%; Chi-square test: P-value = 0.0044).

The negative citation is more frequent among retracted manuscripts reporting an US diagnostic method (4.84%) as compared to manuscripts reporting other diagnostic methods (0.32%, P-value = 0.0004).

## Discussion

Post retraction citations proved to be a current practice among articles reporting radiology diagnostic techniques, and in some cases with more post retraction citations compared to citations prior to the retraction. Furthermore, in most cases, the notice of retraction did not appear either in the text or in the reference list, with a low percentage of negative citations.

The retraction of articles reporting results related to a radiology diagnostic method remains low (3.5%), but compared to the previously reported results, the values increased almost threefold (1.1% [15]). This result is to be expected, since the number of published articles increases, and it is similar with the pattern observed among other medical disciplines (neurosurgery [31], orthopedic research [32], cancer research [14], biomedical Open Access literature [33]).

The original articles were the most frequent type of retracted manuscripts, followed by case reports (Table 1), reflecting the trend in radiology scientific publications. The majority of evaluated articles were published in a journal with an impact factor (Table 1), i.e. journals with a rigorous editorial process. However, the rigor of the editorial process could explain the high number of retracted articles. Half of the retracted articles were retracted in one year, the period of time being double when only articles reporting an US technique were considered (Table 1). The time interval between publication and retraction is shorter as compared to the median of 2 years reported by Rosenkrantz [16], and longer for the retracted papers that reported an US diagnostic method (Table 1). Most of the articles (20) are retracted within the first 12 months (20 articles) after publication, with most of them being retracted in the first two years (Fig 2B).

The top three retraction reasons (Table 1) in our study were similar to those already reported in the scientific literature. Grieneisen and Zhang reported that the main reason for a retraction is the publishing misconduct, primarily plagiarism and author-initiated duplicate publication (47%), closely followed by research misconduct motivated by questionable data or interpretations, legitimate artifacts, or re-interpretation of conclusions in the light of new facts (42%) [7]. Different reasons for retraction are observed in a retracted paper that reports an US diagnostic method as compared to non-US retracted articles, with a smaller number of retractions for error in data/manuscript, publisher error, or the absence of co-authors consent (Table 1).

Based on this result, a different pattern explained by both researchers and/or editorial policies regarding different radiology diagnostic methods is expected and needs further investigation.

The majority of the authors of the retracted manuscripts were from Germany, closely followed by USA, Romania, France, Italy, and Netherlands. The highest number of retracted articles reporting an US method occurs mainly in Italy, India, Germany, and Croatia, shaping a slightly different pattern. These distributions could be a reflection of the main radiology research centers, of the constraints for publication in academia, or of the dynamic of diagnostic imaging fields.

The WoS domains of the investigated retracted articles are more than Radiology, which is probably because the results regarding the radiology-imaging diagnostic methods could be part of a therapeutic or prognostic study and the interdisciplinarity of research is frequently observed in biomedical studies. The *American Journal of Roentgenology* has with the lowest retraction rate while, the *Diagnostic Pathology* has the highest retraction rate among the journals indexed in WoS. This result could be explained by the thoroughness of pre- or post-publication processes of those journals.

Throughout the sample, the retracted articles received more citations post retraction compared to the number of citations prior to the retraction, with few exceptions (Fig 3A). Furthermore, the pattern of prior and post retractions among papers that reported an US method is slightly different (Fig 3C). Different patterns are observed also when the weighted citation index is analyzed, with smallest values in most of the cases for the retracted manuscript that reported an US method (Fig 3D). The post retraction citation appears mostly in papers with a single country affiliation of the authors, with a median of five authors, and is clearly noticed in the text of the manuscript or in the reference section only in a very small number of cases (6 cases, Table 2). A negative citation of a retracted manuscript is observed in 13 cases, 12 of them belonging to a retracted article that reported an US method (Table 2).

The weighted citation index (WCI), considering a ponderation with the citation window, showed no significant pattern among articles reporting an US diagnostic method compared to other diagnostic methods, neither prior to nor post retraction (P-values>0.5). However, the WCI_prior_ showed a slightly higher value for the manuscripts reporting other radiology diagnostic methods than US (median = 0.87) as compared to the manuscripts reporting an US diagnostic method (median = 0.81) and a slightly higher WCI_prior_/WCI_post_ score (1.7 for the manuscript reporting other radiology diagnostic methods than US vs. 1.5 manuscript reporting an US diagnostic method). A tendency to a significant WCI post retraction compared to WCI prior to the retraction is observed just for the papers dealing with an US diagnostic method (P-value = 0.08, the value of the WCI_post_ third quartile decrease almost three times as compared to WCI_prior_). The absence of significant differences between WCI_prior_ and WCI_post_ showed that the post retraction citations continue to exist even if it is expected either to significantly decrease, to be negative or to clearly state that the article was retracted.

A retracted article received citations up to 21 years with more than 10 citations in the first six years after retraction (Fig 4). The high number of citations received in the same year as retraction could be explained by the absence of retraction prior to the acceptance of the citing manuscript. The same explanation could also be appropriate for the citation received in the first two years after retraction as the time period from submission to retraction could be long and varies from one journal to another.

More retracted papers reporting an US diagnostic method received negative citations (4.84%, P-value<0.05), but the number remains very small.

The continued use of retracted articles as a reliable source of information has previously been reported and this behavior could be explained by the low visibility of the retraction notices [34]. Even when a manuscript appears as retracted, the retraction notice is not easily accessible and sometimes requires proficient search skills. The researcher might need to read the retraction notice from the journal page. Therefore, it is essential to make the retracted articles transparent, visible and clear to readers in order to avoid post retraction citations. A standardized policy for reporting article retractions should be generally accepted as a valuable tool for a consistent and transparent informing method. The standardized policy regarding the citation of a retracted article could avoid the large discrepancy that exists between journals concerning the announcement of retraction (some journals offer details about the reasons for retraction, whilst others provide only a retraction statement without any justification for retraction). Furthermore, the research team should carefully check the references prior to publication using the bibliographic database resources, avoiding the second-hand citation (citation of an article cited by another article). In this way, the previous articles cited in more recent articles are checked to establish if they are still accurate, not retracted and if the provided information is still valid. Misconduct, error, or unethical research are serious sources of errors in a retracted article and could interfere with the validity of findings, especially when such articles are used to substantiate a new study or in systematic reviews and meta-analyses. How retractions are handled by journals and how to improve the rules of conduct regarding article retractions still remains an ethical problem, which could exert a positive or negative influence on the researchers and their results. Elia at al. showed that guidelines for retracting articles are incompletely followed, and the retraction process needs to be clarified (only 6% of the retracted article were found to be adequately retracted) [35].

To add a retracted article on the reference list can lead to a potential chain reaction of repeating the same error as in the retracted article. Therefore, the responsibility for citing a retracted article belongs first to the authors, but the identification of the reason for retraction could be problematic and, in most cases, time-consuming. Although the authors are responsible for the validity of information in their scientific manuscripts, biomedical librarians are in an unique position to notify the retracted papers [36]. Several studies showed that articles having a high number of citations prior retraction are more likely to receive additional citations post retraction [37,38] and this can be explained by a second-hand citation behavior (citing an article previously cited by another article) in the absence of reading the primary source of the cited manuscript.

### Ethical considerations and call for solutions

In academia, retracting a published paper is perceived as a sanction for the author(s). However, in most cases, the retraction *per se* does not produce any consequence. Due to the many and different reasons for retractions, the ethical ramifications are complex. Institutional administrative measures should be undertaken in case of misconduct or fraud (intentionally), and the sanction could be a restitution of funds, removal from the project, additional training, probation, suspension, salary reduction, or initiation of steps leading to possible university rank reduction or ending employment [39]. The reputation of authors and the cause of the retraction (fraud vs. mistake, honest error) „*shape the magnitude of the penalty*”, and the well-known scientists are „*more harshly penalized*” than others, while known or less known authors are treated in the same way when the retraction reason is “*honest mistake(s)*” [40]. Education and training of researchers in the spirit of integrity and honesty should be applied in case of honest error(s) and is mandatory to assure accuracy in science. Institutions should be open to these issues, to improve educational programs for all researchers and mentors and to develop fair and appropriate procedures for handling such trends and facts. Institutions should have an important role in avoiding and correcting the deviations from research integrity; however, in most cases, the institutions remain uninvolved, without any involvement in the retraction process [41,42]. The absence of implication of the host institution in the retraction process could be explained by the lack of experience of ad hoc committees members (which generates confusion in understandings and interpretations), discrepancy between procedures and consequences of their implementation, and by „*the process of sense hiding*” of integrity issues that determines researchers „*to stick to current practices*” [43].

Post retraction citation is a matter of ignorance or lack of research integrity. Citing an article solely on the basis that it has been previously cited is unethical because research integrity mandates referring to those manuscripts read in full text by the authors. Citation of a retracted article as a negative citation is ethical and is encouraged. PubMed introduced the possibility to search for retracted articles but unfortunately did not cover all fields of research and is limited to the indexed journals. The Retraction Watch Database (http://retractiondatabase.org/, Version: 1.0.5.5, accessed January 8, 2019) is a valid solution for checking whether an article is retracted or not, providing the reason for retraction along with the information related to the retracted manuscript and retraction note. To keep the database up-to-date, a worldwide effort is needed, and in this regard, an identified retracted manuscript could be submitted to the retraction watch by following the instructions in the user guide. With the scientific community’s effort and proper scientific integrity training sessions, the post retraction citations should display both the original retracted article as well as the retraction notice, with clear notification in the reference list that the article was retracted.

### Study limitations

The search for the retracted articles was conducted with PubMed Clinical Queries that offer the possibility to search for the retracted articles in a standardized way. Searching using only one database is one of the primary limitations and, thus some of the retracted articles reporting a radiology diagnostic method may have been neglected. The use of other databases such as Web of Science, Science Direct, EM-BASE, EBSCO, JSTOR, etc. may produce a more comprehensive view related to the retraction of radiology diagnostic method papers.

The number of articles included in our analysis remained low compared to other similar studies of retracted publications on biomedical sciences, and thus, the conclusions drawn from the results are limited.

The evaluation of articles published in English is another important limitation of our study. A more exhaustive search that also includes articles published in Chinese (Chinese Social Sciences Citation Index database), Spanish (SCIELO database), or Russian (Russian Science Citation Index database) may produce different and more comprehensive results.

In addition, the main reason for retraction was collected, but an article could be retracted for more than one reason. The evaluation of all reasons for retraction may provide a more realistic view. Furthermore, the evaluation of the citation excluded 22% of the identified manuscript due to the unavailability of the full text. The evaluation of all papers citing a retracted manuscript may lead to a more accurate picture regarding positive/negative citation of the retracted manuscript involving radiology diagnostic techniques.

## Conclusions

Our study provides the first insights regarding the post retraction citations in radiology-imaging diagnostic methods scientific literature available in PubMed. The retractions are mainly due to plagiarism, repetitive publication and error in data. The post retraction citations are twice as frequent for the whole sample of analyzed manuscripts but are halved for those manuscripts that report an ultrasound diagnostic method. Concerning the citation window (WCI), there was no significant difference between the number of citations before (WCI_prior_) and after (WCI_post_) retraction. In less than 2% of cases, the retracted manuscript appears as retracted in the reference list or in the text of the manuscript and in less than 5% the citation of a retracted manuscript is negative.

Obligatory training of the medical researchers with regards to academic integrity is now required within academic programs, and the involvement of institutions in this process is mandatory. The editors of medical journals must also educate the authors not to refer a retracted article in their papers or when they choose to do so, to clearly indicate the retraction status of the paper and to cite both the original paper and the retracted notice.

## Supporting information

S1 File Raw data of articles included in the analysis and characteristics of the post retraction citations. (XLSX)
